# Revisiting Catalytic Methods Exploiting Digital Videos: Sequential Determination of Copper and Sucrose in Sugarcane Spirits

**DOI:** 10.1021/acsomega.5c07149

**Published:** 2025-11-10

**Authors:** Gabriel M. Fernandes, Fábio R. P. Rocha

**Affiliations:** Center for Nuclear Energy in Agriculture, University of São Paulo, Av. Centenário, 303, Piracicaba, São Paulo 13416-000 Brazil

## Abstract

Catalytic methods are a branch of sensitive analytical methods that demand strict time control to achieve reliable results. The present work is a pioneering exploration of digital videos recorded by a smartphone camera to accomplish catalytic methods, herein with a focus on the determination of Cu­(II) and sucrose in sugarcane spirits. In an alkaline medium (pH > 12.4), the metal ion catalyzes the air oxidation of 1,5-diphenylcarbazide (DPC, colorless) to 1,5-diphenylcarbazone (DPCO, orange) and further to 1,5-diphenylcarbadiazone (DPDO, colorless). Furthermore, the catalytic effect is inhibited due to the complexation of Cu­(II) by sucrose. Under proper adjustment of alkalinity, both analytes were determined from kinetic curves obtained by extracting the channel *G* values (RGB color system) from the videos. Under optimized conditions, linear responses were obtained from 0.06 to 0.95 mg L^–1^ Cu­(II) and 0.1 to 5.0 g L^–1^ sucrose (*r* ^2^ > 0.99) with an LOD of 0.02 mg L^–1^ Cu and 0.03 g L^–1^ sucrose, with coefficients of variation of 4.5 and 4.2% (*n* = 10), respectively. Recoveries from 91 to 108% Cu­(II) and 95 to 118% sucrose demonstrated the absence of a matrix effect, and accuracy was assessed in relation to reference FAAS and Lane–Eynon methods. The proposed catalytic method is a cost-effective and environmentally friendly alternative for quantifying these important quality control parameters in sugarcane spirits, requiring only 60 μg of DPC and 2 mg of NaOH, with 1 mL of waste generated per determination.

## Graphical abstract

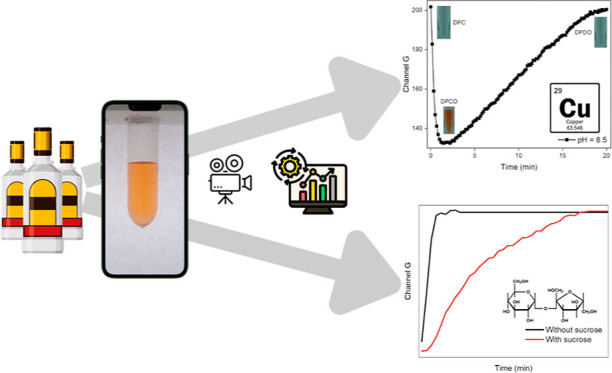

## 1 Introduction

Catalytic analytical methods are a class of kinetic methods that exploit the effect of the analyte on the rate of an indicator reaction.^1^ These methods typically exhibit high sensitivity, sometimes reaching limits of detection (LOD) on the order of nmol L^–1^.^2^ They encompass a variety of reactions, such as redox, ligand-substitution, and metalloporphyrin formation, as well as a diversity of analytical applications. However, the need for strict time control in kinetic methods impairs the widespread application, and most catalytic methods rely on flow-based systems.^3^ A search on the Scopus and Web of Science databases evidenced a significant decrease in the number of publications of catalytic methods in the last 15 years, showing that practical alternatives to revisit this approach would be welcome.

The use of smartphone-based digital images (SDI) is rapidly expanding in areas such as food, environmental, and clinical analysis,^4^ due to their cost-effectiveness, portability, and user-friendliness. The methods are typically more environmentally friendly, owing to the low energy demand and capability for miniaturization with minimal reagent use and waste generation. Another perspective is to explore digital videos for kinetic monitoring, as demonstrated for the detection of matrix effects^5^ and the monitoring of a gas diffusion extraction.^6^ Digital videos were also successfully exploited for monitoring the reaction rate in enzymatic methods, which was applied for ethyl carbamate determination in sugarcane spirits based on its inhibitory effect on acetylcholinesterase activity.^7^ Furthermore, digital videos have been applied for determination of reaction order^8^ and evaluation of the reaction kinetics of colorimetric methods for copper, iron, and phosphate determination based on reactions carried out in reagent drops.^9^

Obtaining reliable results by SDI depends on the suitable selection of the color system, considering the analytical response, selectivity, and linearity.^4^ The RGB color system, based on the combinations of red, green, and blue components, is the most common for analytical applications,^10^ but grayscale, CMY (cyan, magenta, yellow), HSV (hue, saturation, value), and Lab color systems have also been used.^6^ In smartphones, Bayer filters are employed to process the captured image into R, G, and B channels, with values represented on an 8 bit scale (i.e., responses ranging from 0 to 255).^11^ The channel selection is initially guided by the complementary color of the absorbing species, aiming to maximize sensitivity.^4^ Most applications are based on the reflected radiation, which is inversely proportional to the color intensity.^4^

Sugarcane spirits are distilled beverages produced from fermented sugarcane juices, generally comprising 38–48% (v/v) ethanol,^12^ which are widely produced (7.2 million liters in 2023) and exported worldwide. The beverage quality assurance encompasses the determination of ethanol and higher alcohols, total and volatile acids, esters, aldehydes, and potential contaminants (e.g., copper and lead).^13^

Sugarcane spirits are usually obtained from copper distillers, which improve the organoleptic properties by removing sulfur derivative compounds,^8^ but promote the metal leaching to the spirits, e.g., by the formation of CuCO_3_Cu­(OH)_2_, which is dissolved as Cu­(II) into the final product.^13^ Despite its biological importance, excess copper is toxic and has been associated with neurodegenerative diseases (e.g., Alzheimer’s, Parkinson’s, and prion diseases).^14^ For safety purposes, Cu­(II) needs to be limited to 5 mg L^–1^ according to the Brazilian Ministry of Agriculture and Livestock (MAPA) regulations, and flame absorption spectrometry (FAAS) is recommended as the official method for determination.

Sucrose is usually added to adjust the sensory characteristics of sugarcane spirits, and MAPA established threshold limits of 6 and 30 g L^–1^ for sugarcane spirits and sweetened cachaça, respectively. Its determination is usually carried out by laborious and time-consuming analytical methods, such as high-performance liquid chromatography (HPLC) with refractive index detection,^15^ spectrophotometry after acid hydrolysis,^16^ and redox titration based on the Lane–Eynon method.^17^ While refractive index detectors are less usual in HPLC, the latter approaches involve intense sample heating.

In this work, catalytic methods were revisited, pioneering the use of digital videos. The catalytic effect of Cu­(II) on the oxidation rate of 1,5-diphenylcarbazide (DPC) was exploited for the metal ion determination, whereas the inhibition of the catalytic effect by sucrose, due to complex formation with Cu­(II), was exploited for the indirect determination of the sugar. Both the catalytic effect and its inhibition were measured from the time to achieve the reaction steady state, which was explored for analytical purposes.^7^

## 2 Experimental Section

### 2.1 Samples and Solutions

Deionized water (resistivity >18 MΩ cm), anhydrous ethanol, and analytical-grade reagents were used to prepare the solutions. Sugarcane spirit samples (either from São Paulo or Minas Gerais states, Brazil) were provided by the “Laboratório de Tecnologia e Qualidade de Bebidas” at ESALQ, University of São Paulo (USP), Brazil. The samples contained 39–47%(v/v) ethanol, 8–19 mg L^–1^ total ester, and 6–48 mg L^–1^ volatile acids.

Sucrose (0.1–5.0 g L^–1^) and Cu­(II) (0.06–0.95 mg L^–1^) working solutions were prepared daily by diluting 250 mg L^–1^ and 10.0 g L^–1^ stock aqueous solutions, prepared from copper sulfate pentahydrate (Sigma, Germany) and sucrose (Sigma, Germany). A 0.5 mmol L^–1^ DPC solution was prepared daily by dissolving an adequate amount of 1,5-diphenylcarbazide in deionized water. NaOH solutions of 10 and 100 mmol L^–1^ were prepared from a 2.0 mol L^–1^ stock, prepared by dissolving an adequate amount of NaOH (Sigma, Germany) in deionized water.

### 2.2 Apparatus

The oxidation reaction was performed in 1.5 mL polypropylene microtubes, under video monitoring with a Galaxy S21 smartphone, equipped with a 12-megapixel camera with an f/1.8 aperture lens. To ensure controlled illumination, a general-purpose 50-lx LED lamp was attached to the side of a Styrofoam box (15 cm high, 21.5 cm wide, and 13 cm deep; Figure 1), which had an upper aperture to accommodate the polypropylene microtubes. Videos were recorded at the center of the microtubes with a region of interest (ROI) of 48 × 48. The smartphone was fixed at a 5 cm × 5 cm aperture in the front of the box, 5 cm from the microtubes. Flash and zoom were disabled for video recording.

**Figure 1:**
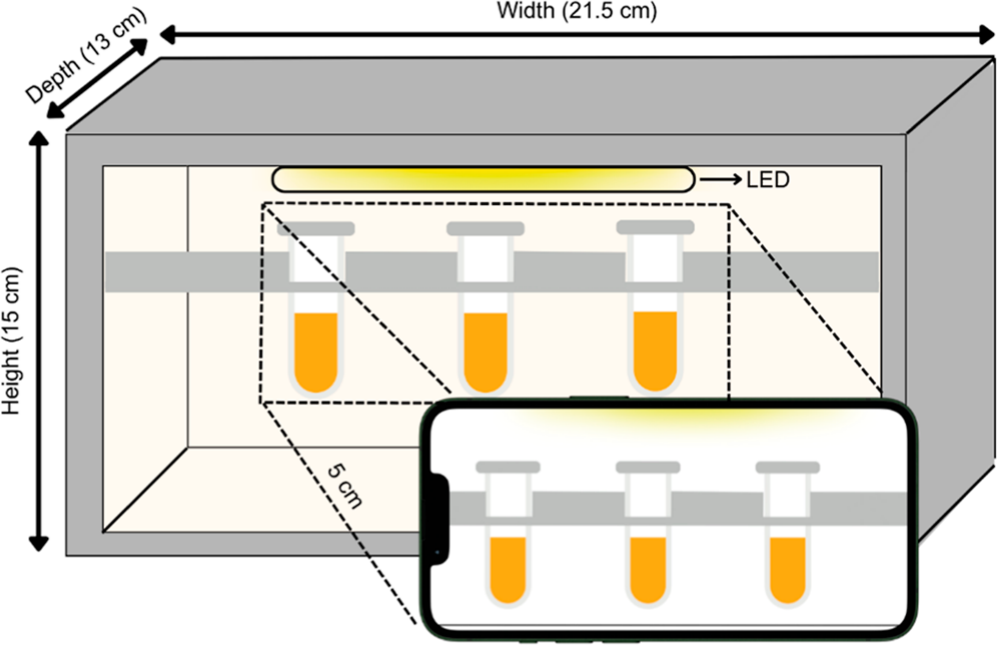
Schematic diagram of the apparatus used to record digital videos under controlled conditions.

Kinetic curves related to either the formation or bleaching of DPCO were obtained by recording digital videos for 15 min. Frames were extracted every 10 s using a custom-made Python script, and channel *G* values (RGB color system) were extracted from the frames using the software ImageJ.^18^

### 2.3 Procedure

In polypropylene microtubes, 250 μL of a sample or reference solution, 500 μL of NaOH, and 250 μL of DFC solution were added. The intensity of the reflected radiation was monitored, as described in Section 2.2. Two different experimental conditions were applied: (I) for Cu­(II) quantification, the NaOH concentration was 10 mmol L^–1^ and the formation of 1,5-diphenylcarbazone (DPCO) was monitored; (II) for sucrose determination, the NaOH concentration was 100 mmol L^–1^ and the bleaching of DPCO was evaluated; this process corresponds to the further oxidation of DPCO to 1,5-diphenylcarbadiazone (DPDO).

The time to reach the reaction steady state (Δ*t*) was measured from the kinetic curves obtained, as described in Section 2.2, and taken as the analytical parameter either directly (sucrose determination) or as 1/Δ*t* (copper determination). All videos were recorded in triplicate at room temperature (25 ± 1 °C).

### 2.4 Reference Procedures

Accuracy for Cu­(II) was assessed based on FAAS (Perkin Elmer spectrometer, model 3110) with calibration by the standard addition method (AOAC 967.08 official method^19^). The operational settings were a current of the hollow cathode lamp of 25 mA, a measurement wavelength of 324.7 nm, and a slit of 0.7 nm. The gas flow rates were 4 L min^–1^ of air and 2 L min^–1^ of acetylene

The AOAC 923.09 Lane–Eynon official method^19^ was adopted for the accuracy assessment of sucrose. Briefly it is based on the acid hydrolysis of sucrose into glucose and fructose followed by a redox titration of the reducing sugars with a Cu­(II) solution under heating. The end point is indicated by the consumption of the methylene blue indicator resulting from the first excess of Cu­(II).

### 2.5 Greenness Assessment

Greenness was evaluated using the AGREE metric,^20^ which scores the 12 principles of green analytical chemistry between 0 and 1 and displays the results in a clock-like diagram. The final score, which is a weighted mean of the 12 parameters, is shown in the center of the diagram.

Sustainability was assessed using the NQS index,^21^ which combines need, quality, and sustainability parameters, evaluated through the need pyramid, the white analytical chemistry algorithm, and the United Nations Sustainable Development Goals, respectively. The final score (0–100%) corresponds to the average of these evaluations.

## 3 Results and Discussion

### 3.1 General Aspects

In alkaline medium, Cu­(II) catalyzes the air oxidation of DPC (colorless) to DPCO (orange) and further to DPDO (colorless),^22^ as indicated in eq 1.
1

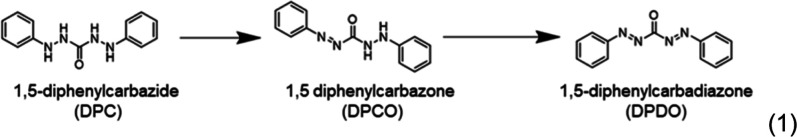




As previously reported,^22^ the oxidation of DPC involves a first-order reaction, as evidenced by the linear log­(slope) versus log­[concentration] plots with correlation coefficients (*R* ^2^) between 0.988 and 0.999. Herein, the first-order behavior was confirmed by plotting the variance of *G* values (sd^2^) as a function of time (*t*) and subsequently plotting ln­(sd^2^) versus ln­(*t*), Figure S1, as previously proposed.^8^ Both plots exhibited asymmetric profiles, which confirms the first-order reaction.^8^

Figure 2 shows the absorption spectra of DPC and its oxidation products, indicating that the reactions can be monitored by either the formation of the orange product (DPCO) or its consumption to yield DPDO, both of which depend on the Cu­(II) concentration in the medium. The latter process occurs relatively quickly at pH 12.4 (a steady state was achieved in less than 10 s under Cu­(II) catalysis, as described in Figure 3A). However, for lower pH (e.g., pH 8.5), oxidation occurs slowly, and monitoring the formation of DPCO is more convenient as described in Figure 3B.

**Figure 2:**
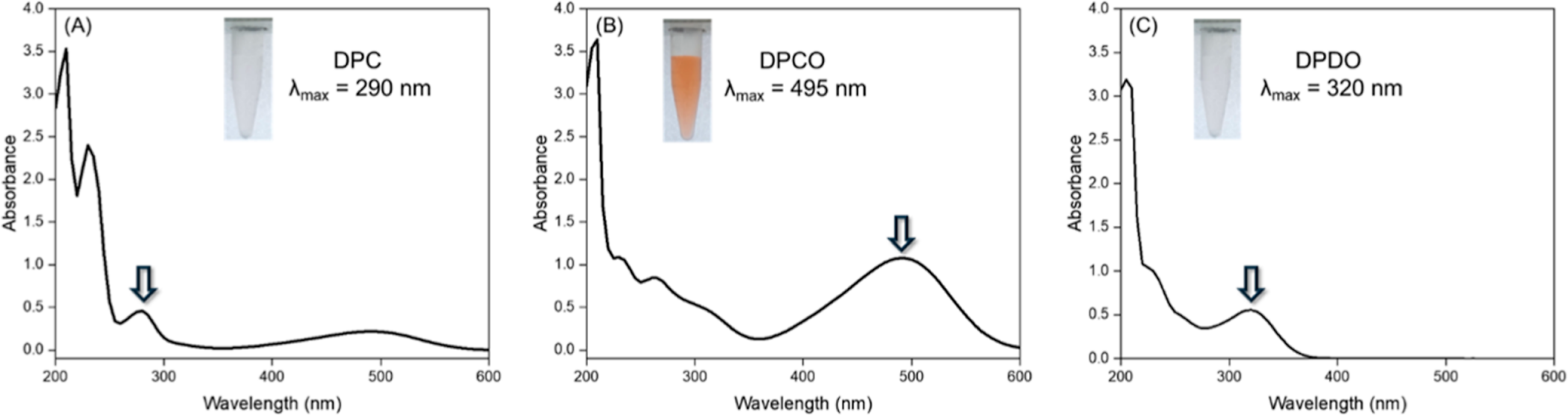
Absorption spectra of 1,5-diphenylcarbazide (A) and its oxidation products (DPCO, B, and DPDO, C) under Cu­(II) catalysis. Reaction conditions: 250 μL of 0.5 mmol L^–1^ DPC, 500 μL of 10 mmol L^–1^ NaOH, and 250 μL of 0.06 mg L^–1^ Cu­(II) solution.

**Figure 3:**
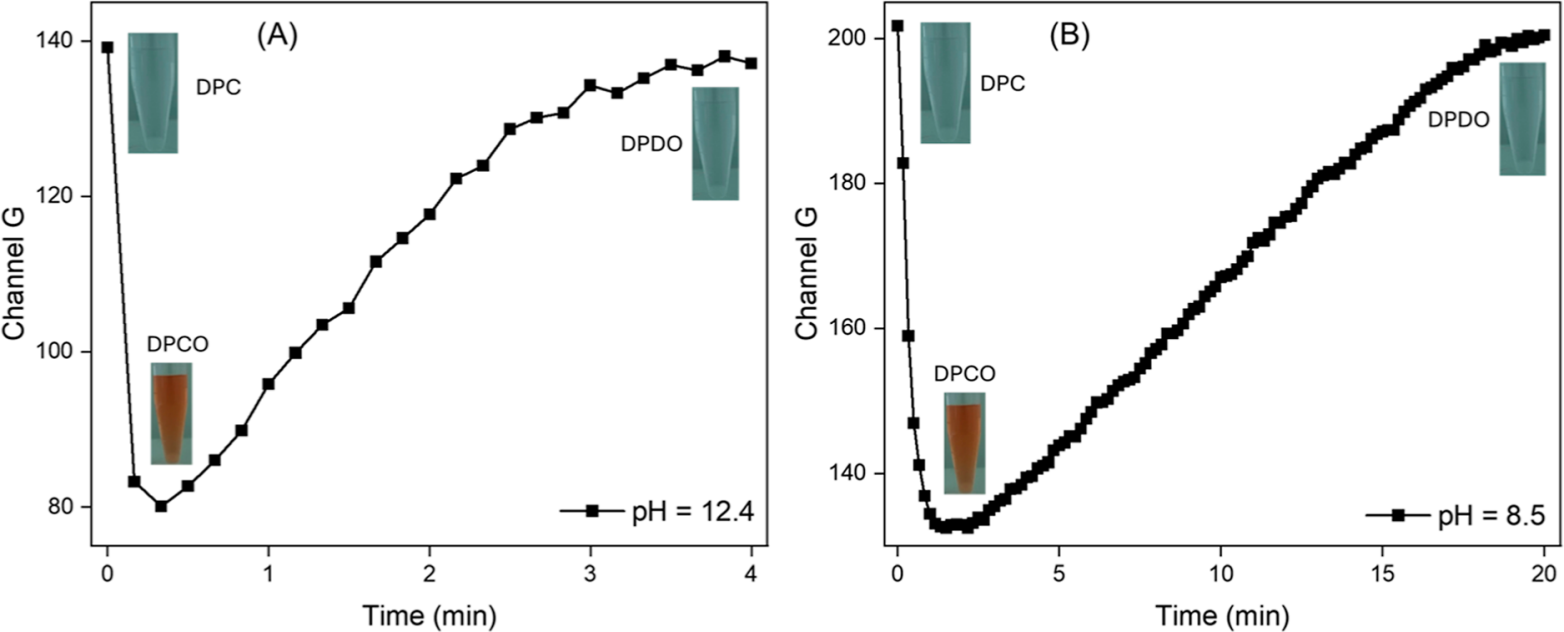
Effect of pH on the catalytic effect of Cu­(II) under the oxidation of DPC monitored by digital videos: 250 μL of 0.5 mmol L^–1^ DPC, 250 μL of 0.32 mg L^–1^ Cu­(II) solution, plus (A) 500 μL of 100 mmol L^–1^ NaOH (final pH = 12.4) and (B) 500 μL of 10 mmol L^–1^ NaOH (final pH = 8.5).

The reactions can be monitored by digital image photometry, and channel *G* (covering the range from 450 to 650 nm) was selected due to its complementarity with the color of DPCO (λ_max_ = 495 nm). In fact, the response in channel *G* accounted for 68% of the total signal variation. As measurements were based on the intensity of the reflected radiation, *G* values were inversely proportional to the color intensity.

Figure 4A shows the catalytic effect of different Cu­(II) concentrations; the increase in *G* values reflects the bleaching of the orange color (oxidation of DPCO to DPDO). The Cu­(II) amount then affects the time to reach the steady state (1.1 and 2.6 min for 0.32 and 0.95 mg L^–1^ Cu­(II), respectively). In alkaline medium (pH > 9), sucrose forms a Cu­(II) complex (*K*_f_ = 2.5 × 10^19^),^23^ inhibiting the catalytic effect on the DPCO oxidation, as shown in Figure 4B. Illustrative videos of the catalytic effect of Cu­(II) and the catalysis inhibition by sucrose are shown in the Supporting Information.

**Figure 4:**
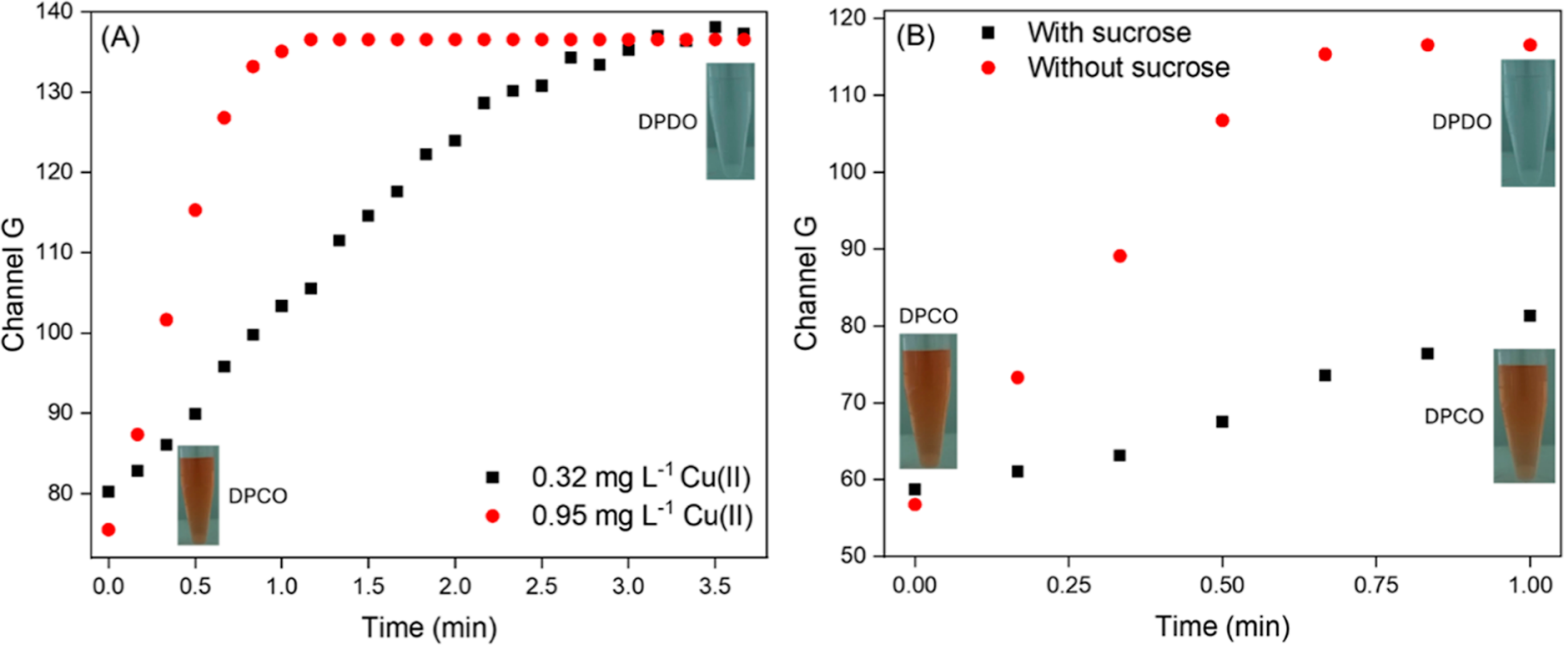
Effect of (A) Cu­(II) concentration and (B) sucrose (2.5 g L^–1^) on the oxidation rate of DPCO at pH 12.4. Experimental conditions: 250 μL of 0.5 mmol L^–1^ DPC, 500 μL of 100 mmol L^–1^ NaOH, and 250 μL of Cu­(II) solution.

The optimal condition for air oxidation of DPC catalyzed by Cu­(II) occurs at pH values above 12.4. However, this high pH also promotes the formation of Cu­(II) complexes with sucrose, which inhibits the catalytic effect. The interference was circumvented by measurements at pH 8.5, which hindered complex formation. Although this lower pH reduces the catalytic efficiency, the analytical response was suitable for the proposed application, as discussed in the following section.

### 3.2 Method Optimization

The univariate method was applied to optimize the experimental conditions, aiming to maximize the catalytic effect of Cu­(II) and its inhibition by sucrose, thereby improving the sensitivity in the catalytic determination of both analytes. Simultaneously, the studies were aimed at minimizing reagent consumption and waste generation. Experimental conditions were initially fixed at 250 μL of sample (either 0.95 mg L^–1^ Cu­(II) or 2.5 g L^–1^ sucrose standard solutions), 500 μL of 100 mmol L^–1^ NaOH, and 250 μL of 0.5 mmol L^–1^ DPC.

Both the oxidation of DPC and the complexation of Cu­(II) by sucrose require an alkaline medium; therefore, different concentrations of NaOH were evaluated to ensure the optimum experimental conditions for each determination. As shown in Figure 5, for a 10 mmol L^–1^ NaOH solution (final pH = 8.5), the effect of sucrose was negligible as complex formation is hindered.^15^ This condition is then suitable for the selective determination of Cu­(II). Conversely, a 100 mmol L^–1^ NaOH solution, which provides a pH = 12.4, maximizes the inhibitory effect of sucrose (0.95 mg L^–1^ Cu­(II) was added to the sample). These conditions were then set for the sequential determination of both analytes.

**Figure 5:**
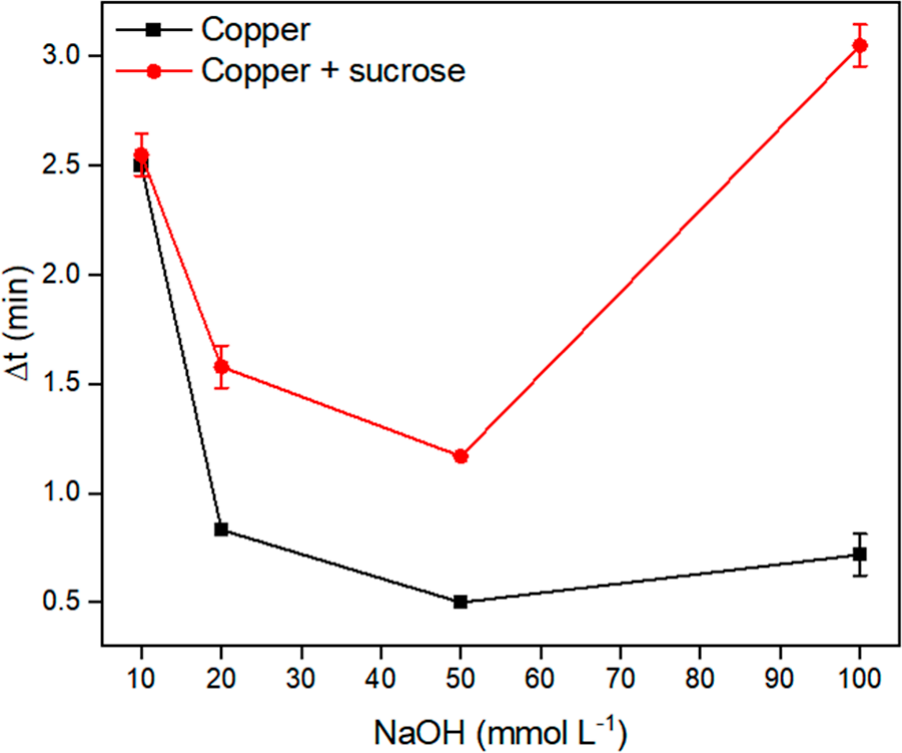
Effect of NaOH concentration on the (■) catalytic effect of 0.95 mg L^–1^Cu­(II) and (⬤) the inhibition of the catalytic effect by 2.5 g L^–1^ sucrose. Experimental conditions: 500 μL of NaOH, 250 μL of 5 mmol L^–1^ DPC, and 250 μL of sample. Data refer to the time to reach the reaction plateau (Δ*t*) in the oxidation of DPCO to DPDO.

Figure 6A shows that Δ*t* increases with DPC concentration in the absence of sucrose, but the effect is moderate, as the experiment was carried out with a relatively high Cu­(II) concentration (0.95 mg L^–1^). In contrast, the inhibitory effect of sucrose, which increases Δ*t*, becomes more evident at higher DPC concentrations (e.g., 500 μmol L^–1^). This is expected, as the amount of the free Cu­(II) catalyst is constant, while the DPC amount increases.

**Figure 6:**
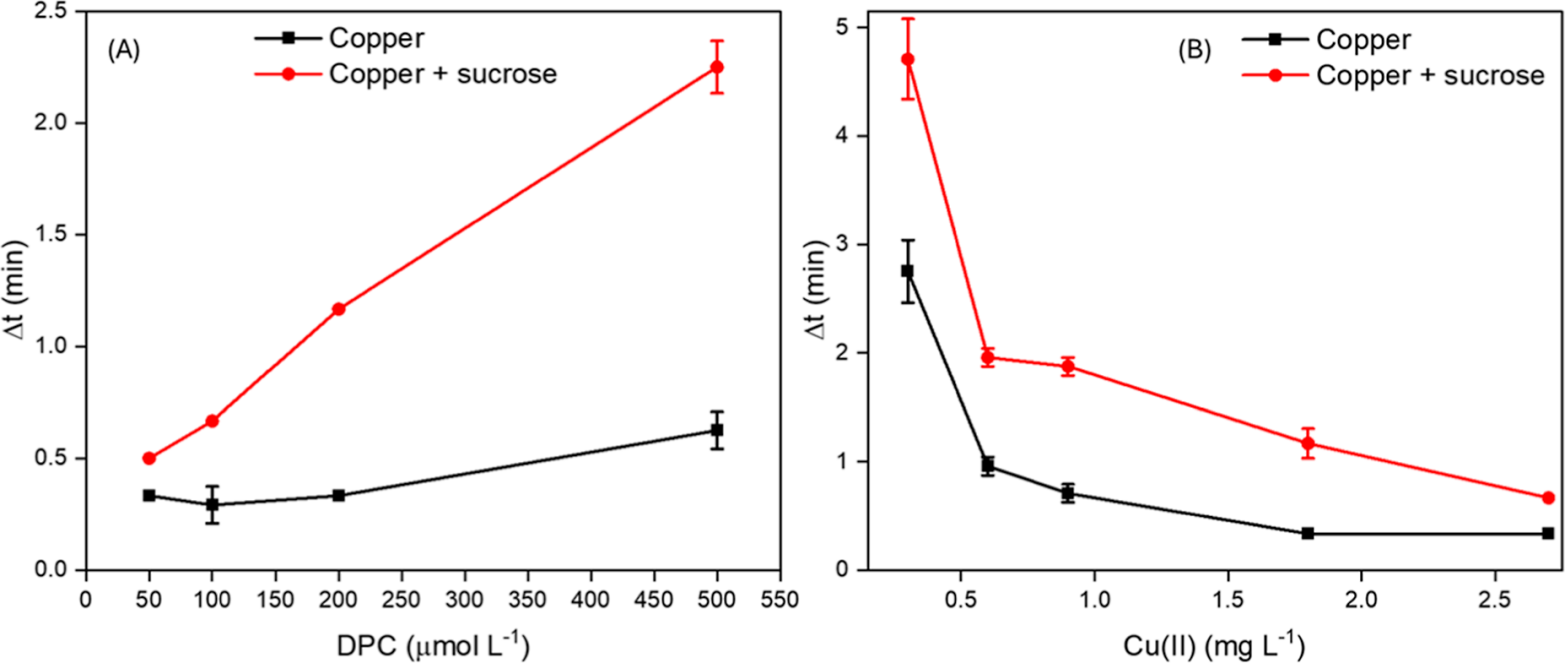
Effect of (A) DPC and (B) Cu­(II) concentration on (■) catalytic effect of Cu­(II) and (●) the inhibitory effect of sucrose. Experimental conditions: 500 μL of 100 mmol L^–1^ NaOH, 250 μL of DPC, and 250 μL of 2.5 g L^–1^ sucrose. (A) 0.95 mg L^–1^ Cu­(II) and (B) 500 μmol L^–1^ DPC. Data refer to the time to reach the reaction plateau (Δ*t*) in the oxidation of DPCO to DPDO.

A significant catalytic effect of Cu­(II) on DPC oxidation is observed up to 0.95 mg L^–1^ with the corresponding diminution of the oxidation time (Figure 6B). The inhibitory effect of sucrose diminished by increasing Cu­(II) concentration, as observed by the difference in Δ*t* values with and without sucrose. Aiming to achieve higher sensitivity with an adequate analysis time, we set 0.95 mg L^–1^ Cu­(II) for further experiments.

### 3.3 Analytical Features and Application

At pH 8.5, 1/Δ*t* varies linearly with Cu­(II) concentration from 0.06 to 0.95 mg L^–1^ of Cu­(II): 1/Δ*t* = 1.53C + 0.001, *R* ^2^ = 0.993, where Δ*t* is the time (min) to reach the plateau of DPC oxidation to DPCO and C is the Cu­(II) concentration (mg L^–1^) (Figure 7A). The linearity of the calibration curve was assessed by the lack-of-fit test at the 95% confidence level (*F*_calc_ = 2.1, *F*_tab_ = 3.7). The LOD of 0.02 mg L^–1^ was estimated as the lowest Cu­(II) concentration that yielded an analytical signal significantly different from the blank at a 95% confidence level. The coefficient of variation (*n* = 10) was 4.5%, and slopes from calibration curves obtained on different days (*n* = 3) were used to assess the reproducibility (interday precision), yielding a value of 6.8%.

**Figure 7:**
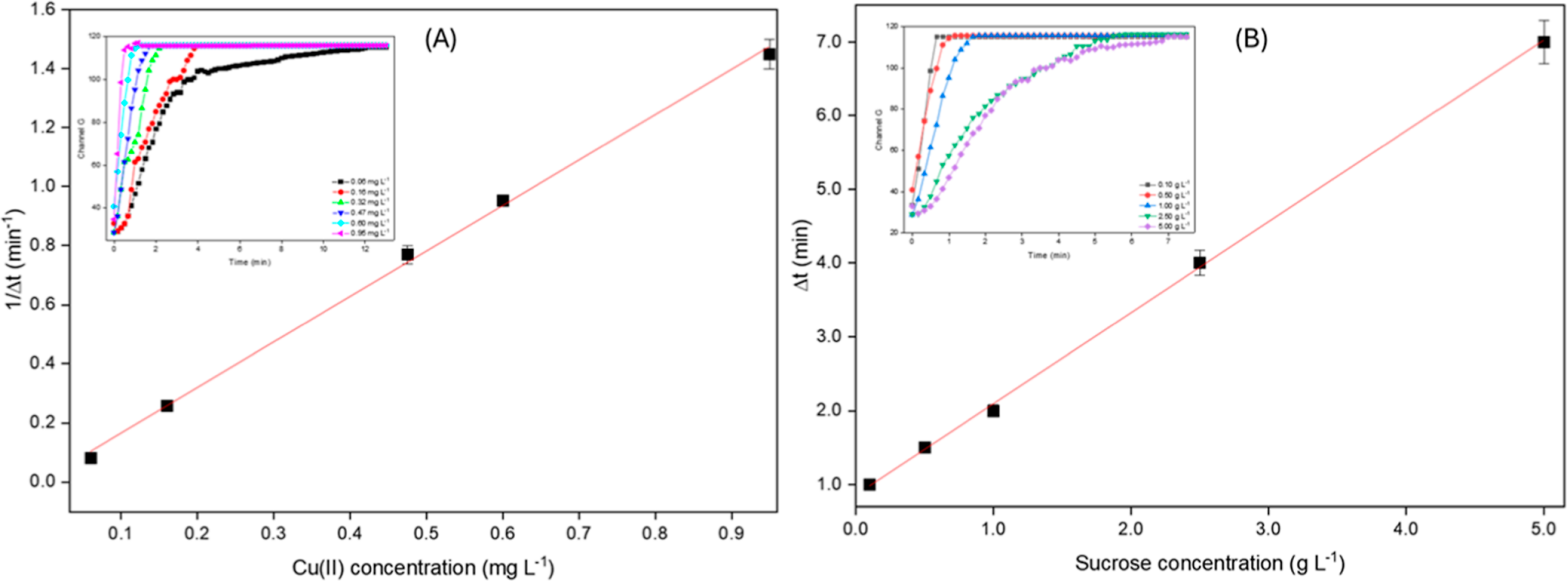
Calibration plots for the catalytic determination of (A) copper and (B) sucrose. Δ*t* represents the time to reach the reaction steady state in the oxidation of DPC to DPCO (A) and of DPCO to DPDO (B). The insets show reaction profiles (channel *G* vs time) for the different analyte concentrations used to obtain the calibration curves.

At pH 12.4, the response for sucrose was linear from 0.1 to 5.0 g L^–1^: Δ*t* = 1.23C + 0.85, *R* ^2^ = 0.999, where Δ*t* refers to oxidation of DPCO to DPDO (time interval in minutes) and *C* is given in g L^–1^ (Figure 7B). Linearity was assessed by the lack-of-fit test at the 95% confidence level (*F*_calc_ = 2.5, *F*_tab_ = 3.5). The LOD of 0.03 g L^–1^ was estimated for sucrose; the coefficients of variation were 4.2% (intraday precision, *n* = 10) and 5.5% (interday precision, *n* = 3).

Table 1 shows the recoveries of the analytes spiked to sugarcane spirit samples, demonstrating the absence of matrix effects as well as the feasibility of measurements at pH 8.5 to avoid the sucrose interference on Cu­(II) determination. However, aged sugarcane spirits exhibited poor recoveries due to the high concentration of phenolic compounds, which can both reduce and form complexes with Cu­(II), leading to a positive error in the sucrose determination.

**Table 1:** Recoveries of Copper and Sucrose Spiked to Sugarcane Spirits (*n* = 3)

	copper (mg L^–1^)			sucrose (g L^–1^)		
sample	added	found	recovery(%)	added	found	recovery (%)
1		0.13 ± 0.01			0.28 ± 0.01	
	0.16	0.28 ± 0.01	94 ± 3	1.0	1.50 ± 0.03	122 ± 2
	0.32	0.54 ± 0.02	128 ± 5	2.5	3.1 ± 0.1	112 ± 4
2		0.23 ± 0.01			0.3 ± 0.1	
	0.16	0.38 ± 0.01	94 ± 2	1.0	1.2 ± 0.1	90 ± 7
	0.32	0.55 ± 0.02	100 ± 4	2.5	2.7 ± 0.1	96± 3
3		0.10 ± 0.01			1.22 ± 0.03	
	0.16	0.26 ± 0.01	100 ± 4	1.0	2.40 ± 0.07	118 ± 3
	0.32	0.42 ± 0.02	100 ± 5	2.5	3.6 ± 0.2	95 ± 5
4		0.41 ± 0.01			0.15 ± 0.01	
	0.16	0.57 ± 0.01	100 ± 2	1.0	1.2 ± 0.1	105 ± 8
	0.32	0.75 ± 0.02	106 ± 3	2.5	2.8 ± 0.1	106 ± 4

FAAS and AOAC Lane–Eynon reference methods^19^ were used to assess the accuracy of the proposed catalytic methods. For copper, results obtained by the proposed and FAAS methods (Table 2) showed normally distributed differences and comparable variances. The results agreed at a 95% confidence level (paired *t*-test: *t*_calc_ = 1.11, *t*_critical_ = 2.57, *n* = 6). Similarly, for sucrose, results agreed with the AOAC Lane–Eynon method (Table 3) at a 95% confidence level, with (*t*_calc_ = 0.41 and *t*_critical_ = 2.57, n = 6). All of the analyzed sugarcane spirit samples met the established limits for copper and sucrose.

**Table 2:** Copper Determination by the Proposed and FAAS^19^ Methods^a^ ^,^ ^b^

sample^c^	proposed (mg L^–1^)	reference (mg L^–1^)	Er (%)	*F*-value
1	0.90 ± 0.04	0.8 ± 0.1	+12	6
2	1.5 ± 0.1	1.6 ± 0.1	–6.3	1
3	1.2 ± 0.1	1.3 ± 0.1	–7.7	1
4	4.0 ± 0.2	4.1 ± 0.3	–2.4	2
5	0.95 ± 0.05	0.90 ± 0.05	+5.5	1
6	2.2 ± 0.1	2.3 ± 0.1	–4.3	1

a Mean values and standard deviations, *n* = 3.

b Er: relative error.

c Samples were 5-fold diluted to fit the method’s linear range.

**Table 3:** Accuracy Assessment for Sucrose Determination in Sugarcane Spirits^a^

sample	proposed (g L^–1^)^b^	reference^c^ (g L^–1^)^19^	Er (%)
1	6.1 ± 0.3	5.8 ± 0.1	+5.2
2	6.4 ± 0.2	6.2 ± 0.1	+3.2
3	16.0 ± 0.7	18.0 ± 0.2	–11.1
4	16.0 ± 0.8	15.0 ± 0.1	+6.6
5	5.8 ± 0.2	5.4 ± 0.1	+7.4
6	17.0 ± 0.6	18.0 ± 0.2	–5.5

a Mean Values and Standard Deviations, *n* = 3.

b Samples were 5-fold diluted to fit the method’s linear range.

c Coefficients of variation, typically <1%, Er: relative error.

Table 4 presents a comparative evaluation of the catalytic method in relation to previously proposed approaches, including a greenness assessment using the AGREE metric.^20^ The proposed method shows suitable sensitivity and selectivity for the proposed application and requires few reagents and a simple apparatus, achieving an AGREE score of 0.72. The official FAAS method for copper determination^24^ requires calibration by the standard addition method to circumvent matrix effects, making it more labor-intensive and impacting the sample throughput (AGREE = 0.65). The digital image fluorescence method^25^ presents a 10-fold higher LOD (0.2 mg L^–1^) and involves the labor-intensive synthesis of carbon dots, which leads to a low AGREE score (0.57). The electrochemical method^26^ requires high sample volumes compared to the proposed method, and internal standard calibration is needed to circumvent matrix effects.

**Table 4:**
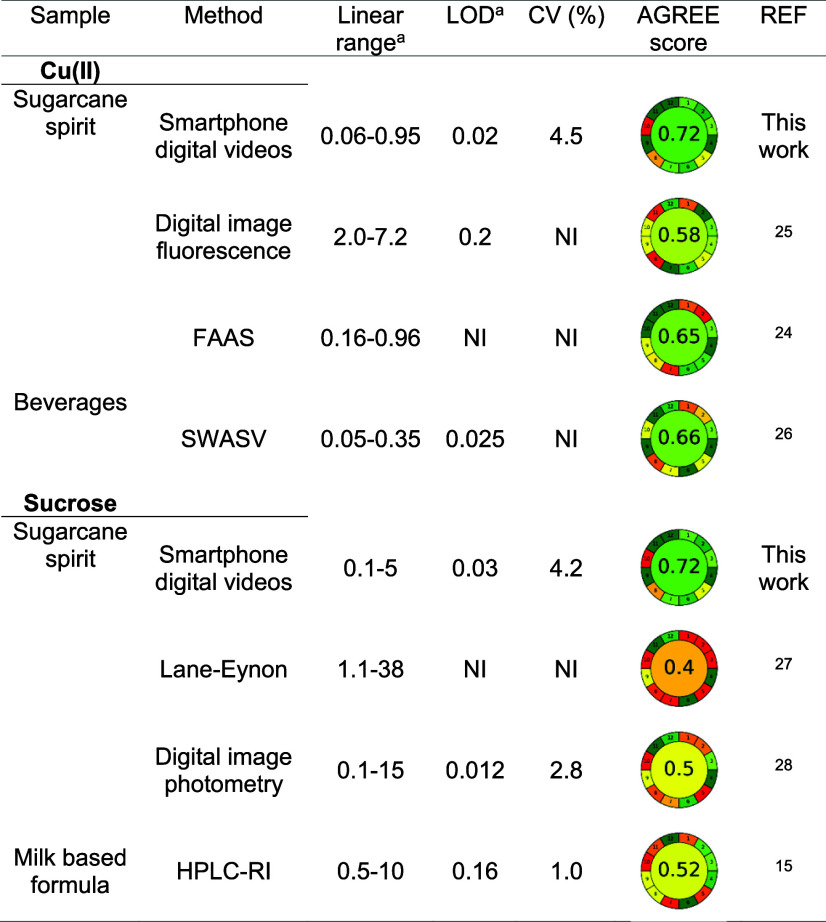
Analytical Features and Greenness of Analytical Methods for the Determination of Copper and Sucrose in Beverages and Milk

a mg L^–1^ for copper and g L^–1^ for sucrose. NI: not informed; SWASV: Square wave anodic stripping voltammetry.

The proposed method is also an attractive alternative to sucrose quantification without the hydrolysis step, which makes it simpler and greener (AGREE = 0.72) when compared to the Lane–Eynon^27^ and digital image-based^28^ methods. The official titration method for sucrose determination is labor-intensive and uses high sample volumes and intensive heating, presenting a low sample throughput; however, the major drawback is the need for an hydrolysis step of sucrose to yield the reducing sugars (AGREE = 0.4). The digital image method also requires an acid hydrolysis step to enable sugar quantification (AGREE = 0.5), whereas HPLC methods with refractive index detectors^15^ are time-consuming (AGREE = 0.52). The NQS score for both copper and sucrose methods was 65%, which indicates their practicality and sustainability.^21^

## 4 Conclusions

This work demonstrates the feasibility of developing catalytic methods using smartphone-based digital videos as a simple and cost-effective approach. The data extracted from videos enabled the exploration of kinetic reaction aspects, offering insights into catalytic behavior in a practical and accessible way. Reliable results were achieved by measuring the time to reach the reaction steady state; however, different approaches may be adopted, such as measuring the variation of G value at a fixed time.

By exploring digital videos, a novel, green, and user-friendly analytical method was proposed for determining copper and sucrose, which are relevant quality control parameters of sugarcane spirits. Adjustment of the reaction conditions enabled the selective determination of both analytes, thereby contributing to the quality control of sugarcane spirits. Furthermore, the indirect quantification of sucrose avoided the need for the time-consuming hydrolysis step and heating used in colorimetric procedures to determine this sugar. The method is attractive for application in production plants because of its practicality and user-friendly instrumentation.

## Supplementary Material







## Notes

Reaction order evaluation using digital videos (PDF)
Catalytic effect of copper under DPC oxidation (MP4)
Sucrose inhibition effect in DPC oxidation (MP4)

## References

[1] Mottola H. A., Perez-Bendito D. (1994). Kinetic Determinations and Some Kinetic Aspects of
Analytical Chemistry. Anal. Chem..

[2] Chen Z., Zhang N., Zhuo L., Tang B. (2009). Catalytic Kinetic Methods
for Photometric or Fluorometric Determination of Heavy Metal Ions. Microchim. Acta.

[3] Moreno A., Silva M., Perez-Bendito D. (1984). Simultaneous
Spectrofluorimetric
Determination of Iron and Manganese by a Differential Kinetic Catalytic
Method. Anal. Chim. Acta.

[4] Soares S., Fernandes G. M., Rocha F. R. P. (2023). Smartphone-Based
Digital Images in
Analytical Chemistry: Why, When, and How to Use. TrAC - Trends Anal. Chem..

[5] Gonçalves I. C., Fernandes G. M., Rocha F. R. P. (2024). Exploiting Digital Images and Videos
for Urea Determination in Milk Based on Enzymatic Hydrolysis Monitoring. J. Food Compos. Anal..

[6] Fernandes G. M., Barreto D. N., Batista A. D., Petruci J. F. S. (2023). A Fully Integrated
3D Printed Platform for Sulfite Determination in Beverages via Gas
Diffusion Membrane Extraction and Digital Video Treatment. Food Chem..

[7] Fernandes G. M., Alcarde A. R., Rocha F. R. P. (2025). Kinetic
Determination of Ethyl Carbamate
in Sugarcane Spirits Exploiting Digital Videos. Talanta.

[8] Agrisuelas J., García-Jareño J. J., Guillén E., Vicente F. (2020). Kinetics of Surface Chemical Reactions
from a Digital
Video. J. Phys. Chem. C.

[9] Apichai S., Thajee K., Wongwilai W., Wangkarn S., Paengnakorn P., Saenjum C., Grudpan K. (2019). A Simple Platform
with Moving Drops
for Downscaling Chemical Analysis Incorporating Smartphone Detection. Talanta.

[10] Costa R. A., Morais C. L. M., Rosa T. R., Filgueiras P. R., Mendonca M. S., Pereira I. E. S., Vittorazzi B. V., Lyra M. B., Lima K. M. G., Romao W. (2020). Quantification of Milk
Adulterants (Starch, H_2_O_2_, and NaClO) Using
Colorimetric Assays Coupled to Smartphone Image Analysis. Microchem. J..

[11] Fan Y., Li J., Guo Y., Xie L., Zhang G. (2021). Digital Image Colorimetry
on Smartphone for Chemical Analysis: A Review. Measurement.

[12] Miranda M. B., Martins N. G. S., Belluco A. E. D. S., Horii J., Alcarde A. R. (2007). Chemical
Quality of Brazilian Sugarcane Spirits. Chem.
Qual. brazilian sugarcane spirits.

[13] Lima C. M. G., Benoso P., Pierezan M. D., Santana R. F., Hassemer G. S., Rocha R. A., Dalla
Nora F. M., Verruck S., Caetano D., Simal-Gandara J. (2022). A State-of-the-Art
Review of the Chemical Composition
of Sugarcane Spirits and Current Advances in Quality Control. J. Food Compos. Anal..

[14] Barbosa E. R., Machado A. A. C., Cançado E. L.
R., Deguti M. M., Scaff M. (2009). Wilson’s Disease: A Case Report and a Historical Review. Arq. Neuropsiquiatr..

[15] Chávez-Servín J. L., Castellote A. I., López-Sabater M. C. (2004). Analysis of Mono-
and Disaccharides in Milk-Based Formulae by High-Performance Liquid
Chromatography with Refractive Index Detection. J. Chromatogr. A.

[16] Miller G. L. (1959). Use of
Dinitrosalicylic Acid Reagent for Determination of Reducing Sugar. Anal. Chem..

[17] Lane, J. H. ; Eynon, L. Determination of Reducing Sugars by Fehling’s Solution with Methylene Blue Indicator; N. Rodger: London, 1934

[18] ImageJ. https://imagej.net/contribute/citing, Accessed on July, 2025.

[19] AOAC Official Methods of Analysis, 15th ed.; Association of Official Analytical Chemists: Washington, DC, 1990;

[20] Pena-Pereira F., Wojnowski W., Tobiszewski M. (2020). AGREE—Analytical GREEnness
Metric Approach and Software. Anal. Chem..

[21] Kiwfo K., Suteerapataranon S., McKelvie I. D., Meng Woi P., Kolev S. D., Saenjum C., Christian G. D., Grudpan K. (2023). A New Need, Quality,
and Sustainability (NQS) Index for Evaluating Chemical Analysis Procedures
Using Natural Reagents. Microchem. J..

[22] Crespo G. A., Andrade F. J., Iñón F.
A., Tudino M. B. (2005). Kinetic
Method for the Determination of Trace Amounts of Copper­(II) in Water
Matrices by Its Catalytic Effect on the Oxidation of 1,5-Diphenylcarbazide. Anal. Chim. Acta.

[23] Norkus E., Vaškelis A., Vaitkus R., Reklaitis J. (1995). On Cu­(II)
Complex Formation with Saccharose and Glycerol in Alkaline Solutions. J. Inorg. Biochem..

[24] Strunk D. H., Andreasen A. A. (1967). Collaborative Study Using Atomic
Absorption Spectrophotometry
for the Determination of Copper in Alcoholic Products. J. AOAC Int..

[25] Maia M. V., Suarez W. T., Santos V. B., Almeida J. P. B. (2022). Carbon Dots on
Paper for Determination of Cu^2+^ in Sugar Cane Spirits Samples
for Fluorescence Digital Image-Based Method. Microchem. J..

[26] Salinas G., Ibanez J. G., Vásquez-Medrano R., Frontana-Uribe B. A. (2018). Analysis
of Cu in Mezcal Commercial Samples Using Square Wave Anodic Stripping
Voltammetry. J. Electrochem. Sci. Technol..

[27] Lane J. H., Eynon L. (1923). Determination of Reducing
Sugars by Means of Fehling’s Solution
with Methylene Blue as Internal Indicator. J.
Soc. Chem. Indust..

[28] Franco M. O. K. K., Suarez W. T., Santos V. B., Resque I. S. (2021). A Novel Digital
Image Method for Determination of Reducing Sugars in Aged and Non-Aged
Cachaças Employing a Smartphone. Food
Chem..

