# Supplementary material for: Revisiting Catalytic Methods Exploiting Digital Videos: Sequential Determination of Copper and Sucrose in Sugarcane Spirits
Source: ACS Omega. 2025 Nov 10;10(46):55787–94. doi: 10.1021/acsomega.5c07149 (PMC12658826; doi:10.1021/acsomega.5c07149)
Supplement: Supplementary file 1 [file ao5c07149_si_001.pdf]

Revisiting catalytic methods exploiting digital videos: sequential determination of copper and sucrose in sugarcane spirits

Supplementary material

Gabriel M. Fernandes, Fábio R.P. Rocha*

Center for Nuclear Energy in Agriculture, University of São Paulo,

Av. Centenário, 303, 13416-000, Piracicaba, SP, Brazil.

*Corresponding author

E-mail: frprocha@cena.usp.br

Evaluation of the reaction order:

The approach previously described by Agrisuelas et al. [https://doi.org/10.1021/acs.jpcc.9b10689] was adopted to evaluate the reaction order of oxidation of DPC by air. The asymmetric curves in the plots of Figure S1 confirms the first-order reaction.

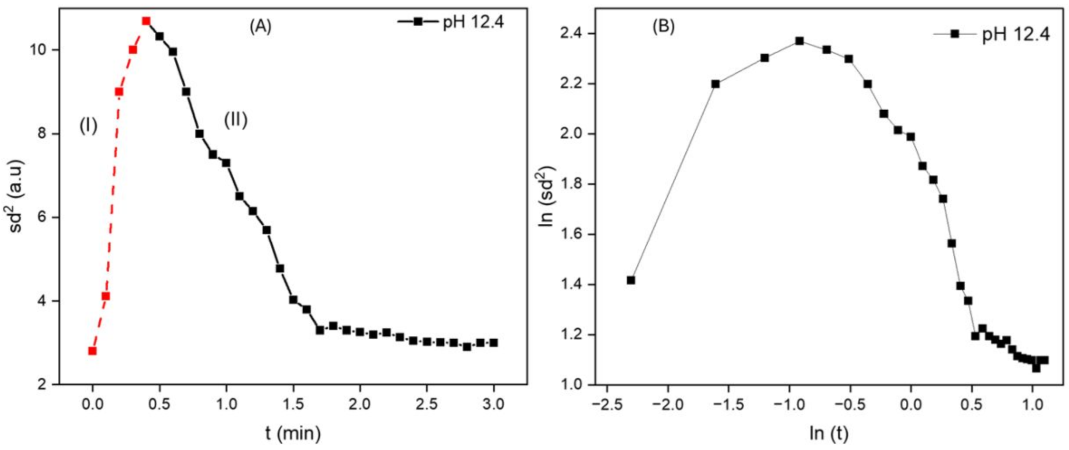

Figure S1. (A) Kinetic profile of DPC oxidation to DPCO (I) and to DPDO (II), sd^2 refers to the variance of G values. (B) Data processing as $\ln(sd^2)$ against ln (t). Experimental conditions: 250 µL of 0.5 mmol L^{-1} DFC, 250 µL of 0.32 mg L^{-1} Cu(II), and 500 µL of 100 mmol L^{-1} NaOH.

Videos

Illustrative videos of the catalytic effect of Cu(II) and the inhibitory effect of sucrose are shown as supplementary material.

Video S1. Oxidation of DPC to DPCO and further to DPDO after adding 250 µL of: (A) 0.06 mg L^{-1} Cu(II) and (B) 0.95 mg L^{-1} Cu(II). Experimental conditions: 250 µL of 0.5 mmol L^{-1} DPC and 500 µL of 100 mmol L^{-1} NaOH.

Video S2. Inhibition of the catalytic effect of Cu(II) by different concentrations of sucrose: (A) 0.1 g L^{-1} and (B) 5.0 g L^{-1}; added volume: 250 µL. Other

concentrations: 0.95 mg L^{-1} Cu(II), 250 µL; 0.5 mmol L^{-1} DPC (250 µL), and 100 mmol L^{-1} NaOH (500 µL). Both videos are shown at 3x speed.
